# Triglyceride to HDL-C Ratio is Associated with Insulin Resistance in Overweight and Obese Children

**DOI:** 10.1038/srep40055

**Published:** 2017-01-06

**Authors:** Nur Ahmad Kamil Zati Iwani, Muhammad Yazid Jalaludin, Ruziana Mona Wan Mohd Zin, Md Zain Fuziah, Janet Yeow Hua Hong, Yahya Abqariyah, Abdul Halim Mokhtar, Wan Mohamud Wan Nazaimoon

**Affiliations:** 1 Department of Paediatrics, Faculty of Medicine, University of Malaya, Kuala Lumpur, Malaysia; 2 Diabetes and Endocrine Unit, Institute For Medical Research, Kuala Lumpur, Malaysia; 3 Department of Paediatrics, Putrajaya Hospital, Putrajaya, Malaysia; 4 Department of Social and Preventive Medicine, Faculty of Medicine, University of Malaya, Kuala Lumpur, Malaysia; 5 Department of Sports Medicine, Faculty of Medicine, University of Malaya, Kuala Lumpur, Malaysia.

## Abstract

The purpose of this study was to investigate the usefulness of triglyceride to hdl-c ratio (TG:HDL-C) as an insulin resistance (IR) marker for overweight and obese children. A total of 271 blood samples of obese and overweight children aged 9–16 years were analysed for fasting glucose, lipids and insulin. Children were divided into IR and non-insulin resistance, using homeostasis model assessment (HOMA). The children were then stratified by tertiles of TG: HDL-C ratio. The strength between TG:HDL-C ratio and other parameters of IR were quantified using Pearson correlation coefficient (r). Odds ratio was estimated using multiple logistic regression adjusted for age, gender, pubertal stages and IR potential risk factors. Children with IR had significantly higher TG:HDL-C ratio (2.48) (p = 0.01). TG:HDL-C ratio was significantly correlated with HOMA-IR (r = 0.104, p < 0.005) and waist circumference (r = 0.134, p < 0.001). Increasing tertiles of TG:HDL-C ratio showed significant increase in mean insulin level (p = 0.03), HOMA-IR (p = 0.04) and significantly higher number of children with acanthosis nigricans and metabolic syndrome. The odds of having IR was about 2.5 times higher (OR = 2.47; 95% CI 1.23, 4.95; p = 0.01) for those in the highest tertiles of TG:HDL-C ratio. Hence, TG:HDL-C may be a useful tool to identify high risk individuals.

Childhood obesity is associated with a wide range of serious health complications and increased risk of premature onset of illnesses, including type 2 diabetes (T2D) and heart diseases. Insulin signalling is impaired in obesity. Impairment of insulin signalling leads to impaired glucose transport, decreased metabolism of adipocytes and skeletal muscle, and increased glucose release by the liver^1^. Measuring insulin resistance (IR), a major cause of T2D, is therefore a useful tool to allow early intervention and prevent or delay the development of the disease. The gold standard method to measure IR is by hyperinsulinemic euglycemic clamp^2^. However, the complexity and high cost of the test has prevented its use in daily clinical practice and in epidemiological studies^3^. Instead, the homeostasis model assessment for insulin resistance (HOMA-IR) index is widely used as a measure of insulin resistance in adults and has also been validated in children and adolescents^4^. However, the HOMA calculations require measurement of plasma fasting insulin and glucose. Due to the unstability of insulin, blood collected for insulin measurement has to be kept cold, immediately processed and plasma frozen as soon as possible. The logistics involved would be a problem especially in field study involving large number of samples^5^. In addition, measuring fasting insulin is not a routine test for obese children. It is clear that there is a need for a diagnostic test for predicting insulin resistance that is easy to carry out, offers good precision and is of low cost.

Triglyceride (TG) and HDL-C on the other hand is a routine test and inexpensive compared to insulin. Currently, lipid profiles can also be tested using portable analyser in clinical setting. Previous studies reported conflicting findings on the usefulness of the triglyceride to HDL-C ratio (TG:HDL-C ratio) as predictor or marker of IR and is ethnicity-dependent^6^,^7^,^8^,^9^,^10^. Triglycerides (TG) are lipids or fats produced by our body as a way to store energy for use. High-density lipoprotein cholesterol (HDL-C) is a beneficial cholesterol molecule that transports fat cholesterol from the body to the liver for excretion or re-utilization. TG and HDL-C are easily and routinely measured in obese patients for a relatively small fee.

In individuals with insulin resistance, TG levels increased while HDL-C levels decreased^11^. Triglyceride to high-density lipoprotein cholesterol (TG:HDL-C) ratio has been proposed to be an alternative tool for gauging insulin resistance^11^. A higher ratio would represent a poorer health status because there is a large amount of circulating fats in the blood stream and/or a low amount of healthy cholesterol. A TG:HDL-C ratio of ≥3 has been shown to be closely correlated to insulin resistance^11^. The TG:HDL-C ratio has also been shown to be an independent risk factor for coronary heart disease (CHD) among Iranian men^12^.

In Malaysia, it was estimated that 3.9% of children under the age of 18 years were obese^13^. A lifestyle school-based intervention programme was therefore developed specifically for overweight and obese children to address this problem. Named as “My Body is Fit and Fabulous (MyBFF@school), the programme was piloted between January and December 2014 in selected public schools in Putrajaya, the Administrative Capital City of Malaysia. The objective of this study was to evaluate the association between TG:HDL-C ratio and IR among the overweight and obese school children who participated in MyBFF@school programme.

## Results

**Figure 1:**
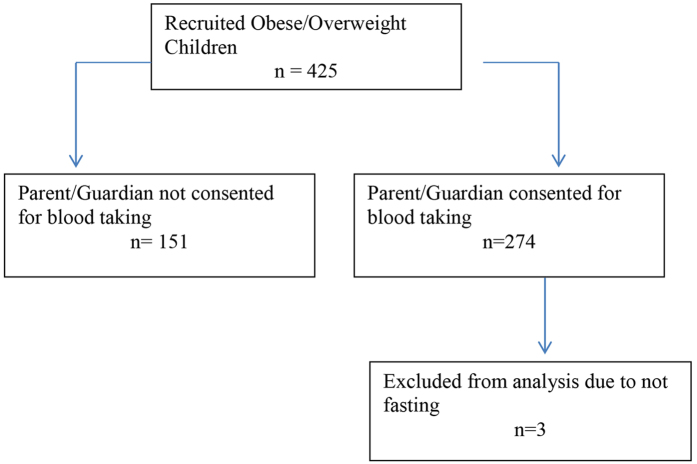
Flow chart for blood data used for TG:HDL-C ratio sub study.

From a total of 425 children who participated, blood samples were obtained from 274; 86 children aged 9–12 years old and 188 adolescents aged 13–16 years old. However, only 271 children had complete data set of lipid values and only their data (n = 271) were used for the analysis (Fig. 1). As shown in Table 1 median age was 14 years old with girls were slightly older than boys (13 years versus 14 years). Gender was equally distributed with 49.1% boys and 50.9% girls. Based on BMI z-score, 22.5% were overweight and 77.5% were obese. A total of 135 (53.1%) children had acanthosis nigricans while 28 (10.3%) children had metabolic syndrome. Table 2 described the general anthropometric and biochemical measurements of the 271 children. There were 126 (49%) children found to have IR as defined by HOMA-IR ≥ 4.0 (Table 3). The mean TG:HDL-C ratio was significantly higher among children with IR compared to the non-IR (2.48 versus 1.73, p = 0.01). Likewise, BMI z-score, waist circumference and body fat percentage were significantly higher in the IR group.

Table 4 summarised the Pearson correlations coefficient (r) between TG:HDL-C ratio and other parameters of IR. TG:HDL-C was significantly correlated with HOMA-IR (r = 0.104, p < 0.005) and waist circumference (r = 0.134, p < 0.001). In addition, HOMA-IR was significantly correlated with BMI z-score (r = 0.202, p < 0.001) and waist circumference (r = 0.258, p < 0.001). Table 5 summarised the anthropometric, clinical and metabolic parameters when stratified according to tertiles of TG:HDL-C ratio. There was no significant difference in age, gender and pubertal status across the tertiles. Highest number of children with acanthosis nigricans (n = 58, 43%) and metabolic syndrome (n = 18, 64.2%) were seen at the third tertiles of TG:HDL-C ratio. However, there was no significant difference in blood pressure status across the tertiles. The BMI z-score and waist circumference rose significantly across TG:HDL-C ratio tertiles (p = 0.03 and p < 0.01 respectively). Similarly, there was significant increase in mean insulin level (p = 0.03) and HOMA-IR (p = 0.04) with increasing tertiles, implying worsening of IR. When based on multiple linear regression analysis using HOMA-IR as the dependent variable and adjusting for age, gender, pubertal stages, waist circumference and BMI-z score, children in the third tertiles of TG:HDL-C ratio posed 2.5 times higher risk of developing IR compared to those in the lower tertiles (OR = 2.47; 95% CI 1.23, 4.95; p = 0.01) (Table 6).

## Discussions

McLaughlin *et al*.^11^ was the first to demonstrate the clinical utility of TG:HDL-C ratio in identifying generally healthy Caucasians with IR. It was shown that the TG:HDL-C ratio was as closely associated with specific measure of insulin-mediated glucose disposal as was the fasting plasma insulin concentration, a surrogate estimate of insulin action that has been widely used to study the relation between IR and various clinical syndromes^11^. However, several studies have shown that the association may be ethnicity dependent. While Hirscheler *et al*.^6^ observed significant association between TG:HDL-C ratio and IR among their indigenous Argentinean children, Gianini *et al*.^7^ showed that the association was only significant among the white obese children but not in the Hispanic or African American children. Similarly, poor association between TG:HDL-C ratio and IR was also reported among adult African–Americans^8^. On the other hand, TG:HDL-C ratio was found to be strongly associated with IR among Korean population^9^ and obese South East Asian Immigrant youths^9^.

In the present study involving overweight and obese children of 9–16 years old, TG:HDL-C ratio was found to be significantly correlated with HOMA-IR (p < 0.05) and was significantly higher (p = 0.01) among children with HOMA-IR ≥ 4. In addition, TG:HDL-C ratio was also found to be significantly correlated with waist circumference (p < 0.001). A number of studies have reported significant predictive power of waist circumference as anthropometric surrogate marker in predicting insulin resistance among children^14^,^15^,^16^,^17^. Hence, indicating the practicality of introducing TG:HDL-C ratio as a relatively simple biochemical marker for IR.

In a study using a large and nationally representative sample (n = 2652) among non-diabetic adults of three major racial/ethnic subpopulations in the United States, Li *et al*.^18^ found that TG/HDL-C ratio was the best predictor of hyperinsulinimia than that of triglyceride or HDL-C alone for the prediction of hyperinsulinemia. In addition, triglycerides and HDL-cholesterol are the two important lipid measures in the diagnosis of metabolic syndrome. A combined lipid ratio may better reflect the overall interaction between lipid/lipoprotein fractions, and therefore associations with insulin resistance^19^.

When stratified according to TG:HDL-C tertiles, we found that mean insulin level and HOMA-IR significantly increased across tertiles. In addition, we also found that significantly higher number of children at the third tertile of TG:HDL-C ratio had acanthosis nigricans and metabolic syndrome. This indicates that besides having significant correlation to HOMA-IR index, TG:HDL-C ratio also have significant association with physical findings (which is very practical) related to the clinical risk of IR.

Moreover, children in the highest tertile of TG:HDL-C ratio posed 2.5 times higher risk of developing IR compared to those in the two lower tertiles. Similar trend was also reported in a study involving indigenous Argentinian children^6^. Indeed, Pacifico *et al*.^20^ also demonstrated that in a study of Caucasian children highest TG:HDL-C ratio showed a 1.8- to 3.8-fold increased risk of central obesity, insulin resistance, high-sensitivity c-reactive protein, non alcoholic fatty liver disease, metabolic syndrome, and increased carotid artery intima-media thickness.

As shown in many studies, hypertriglyceridemia and decreased HDL-C are the hallmarks of dyslipidemia which is a characteristic of insulin resistance and T2D. The Treatment Options for Type 2 Diabetes in Adolescents and Youth (TODAY) study showed that 79.8% of T2D youth had a low HDL-C and 10.2% had high triglycerides within a few months of diagnosis^21^ and the SEARCH for Diabetes in Youth study found that 73% of 2096 US youth with T2D of longer duration had lower HDL and 60–65% had hypertriglyceridemia^22^. Therefore, the clinical utility of measuring TG and HDL-C extended beyond identifying patients with IR. Not surprising, we also found that BMI z-scores and waist circumference rose significantly across TG:HDL-C ratio tertiles. Association between obesity and morbidity, independent of insulin resistance and diabetes has been shown in many studies^23^,^24^,^25^,^26^,^27^. In a prospective study involving general population in China, logistic regression model showed that TG:HDL-C ratio could independently predict future diabetes mellitus^28^.

In conclusion, this study has shown that TG:HDL-C ratio is significantly associated with IR in overweight and obese Malay children. The TG:HDL-C ratio is an inexpensive predictor of IR and may be a useful tool to identify high risk individuals for early intervention and thereby prevent or delay the development of IR-associated diseases such as T2D and hypertension. A larger cohort study involving children from different ethnic groups should be carried out to confirm our current findings.

## Methods

### Study design and population

A total of 425 obese and overweight children aged 9 to 16 years old with body mass index (BMI) z-score exceeded 2 or 3 standard deviation according to WHO BMI chart were recruited for the study (Fig. 1). Children came from a total of 6 randomly selected public schools in Putrajaya, the Administrative Capital City of Malaysia. Exclusion criteria included physical or mental disability that would prevent children from participating in moderate-to-vigorous intensity physical activity and children with known co-morbidities such as T2D, hypertension and cardiovascular disease. Ethical approval was obtained from the Medical Research and Ethics Committee (MREC) Ministry of Health Malaysia. Written informed consent was obtained from parent or guardian and all participating children were also required to sign an assent form. All testing was performed in accordance with the approved guidelines.

### Health and physical examination

Prior to the study visit, these children were asked to fast overnight for at least 8 hours. All anthropometric measurements were performed by trained personnel, and health examinations were performed by pediatricians. Standing height was measured without shoes to the nearest 0.1 cm using calibrated stadiometer (Seca 217, Germany). Body weight and body fat mass were measured in light clothing without shoes and socks to the nearest 0.1 kg using a pre-calibrated body impedance analyzer (InBody 720, Korea). Waist circumference was measured twice to the nearest 0.1 cm over the skin midway between the tenth rib and the iliac crest at the end of normal expiration, using a non-extensible tape (Seca 201, Germany) and the mean was recorded. Two readings of blood pressure was measured after 5 minutes of resting using a mercury sphygmomanometer (Accoson, UK) in a seated position with the arm supported at heart level, and the mean was recorded. Pubertal status was assessed (self-administered) using Tanner staging scale^29^,^30^ and participants were also examined for the presence of acanthosis nigricans over the neck^4^.

### Biochemical parameters

Venipuncture was performed by experienced nurses or doctors. Blood samples were transported cold to the central laboratory at the Institute for Medical Research within 2 hours of collection and processed on the same day. Aliquots of serum/plasma samples were kept at −20 °C prior to analysis. HbA1c level was determined by cationic exchanged high performance liquid chromatography (Adams A1c HA-8160, Arkray Inc, Japan) and followed the National Glycohemoglobin Standardization Programme Guidelines. Fasting plasma glucose, triglycerides, total cholesterol, HDL and LDL were analyzed using an automated analyzer (Dirui CS-400, China) with reagents purchased from Randox Laboratories (Antrim, UK). Fasting insulin concentration was measured using an automated enzyme immunoassay analyzer (TOSOH AIA-360, Japan). Interassay coefficient of variability (CV) for insulin at 8.7, 44.4 and 143.2 μU/ml was 2.5%, 2.6% and 2.4% respectively.

### Definition of Measures

Overweight or obese was defined as BMI z-score exceeded 2 or 3 standard deviation respectively for their age and sex, according to WHO BMI chart^31^. Tanner staging was assessed by showing a standardized Tanner staging pictures to the child where stage 1 external genitalia development and breast development for boys and girls respectively was classified as pre-pubertal, while stage 2 and above was defined as pubertal. Determination of acanthosis nigricans (AN) was based on Burke’s quantitative dichotomous score^4^. Insulin resistance status was based on the homeostasis model assessment (HOMA), calculated by multiplying the value of fasting plasma insulin and fasting plasma glucose and divided by 22.5^4^. The score of ≥4.0 was classified as insulin resistance, while a score of less than 4.0 was considered as insulin sensitive^32^. Metabolic syndrome for children was based on the definition established by International Diabetes Federation (IDF)^33^. Metabolic syndrome was considered present if the waist circumference measurement was ≥ 90^th^ centile of the Malaysian waist circumference chart^34^ with the presence of at least two of the following criteria; triglycerides ≥ 1.7 mmol/L, HDL cholesterol < 1.03 mmol/L, systolic blood pressure ≥ 130 mmHg and/or diastolic blood pressure ≥ 85 mmHg, or fasting plasma glucose ≥ 5.6 mmol/L. Hypertension was defined as systolic and/or diastolic blood pressure of more than 95^th^ percentile for age, sex and height^35^.

### Statistical Analysis

Data analysis was conducted using the IBM Corp. Released 2013. IBM SPSS Statistics for Windows, Version 22.0. Armonk, NY: IBM Corp. and StataCorp. 2015. Stata Statistical Software: Release 14. College Station, TX: Stata Corp LP. Normality test for continuous data was determined using Kolmogorov-Smirnov test. Means and standard deviations (SDs) were calculated for the continuous variables. Comparison of means among groups was conducted using independent group *t*-tests or analysis of variance (ANOVA) where applicable and the Mann–Whitney test was used for not normally distributed variables. Categorical comparisons were made using Chi-square test. Multiple logistic regression analyses were performed to examine the relationship between insulin resistance and TG:HDL-C adjusted for age, gender, pubertal stages, waist circumference and BMI- zscore. All statistical tests were performed at 5% significance level.

## Additional Information

**How to cite this article**: Zati Iwani, N. A. K. *et al*. Triglyceride to HDL-C Ratio is Associated with Insulin Resistance in Overweight and Obese Children. *Sci. Rep.* **7**, 40055; doi: 10.1038/srep40055 (2017).

**Publisher's note:** Springer Nature remains neutral with regard to jurisdictional claims in published maps and institutional affiliations.

## Figures and Tables

**Table 1:** Clinical characteristics of 271 children.

	Boys	Girls	P value	All
N (%)	133 (49.1)	138 (50.9)	NS	271
Age, median (p25, p75)	13 (11, 14)	14 (13, 14)	0.004	14 (11, 14)
**Pubertal status**				
Pre-pubertal (n (%))	46 (37.4)	29 (22.1)	0.008	75 (29.5)
Pubertal (Tanner Stage ≥ 2) (n (%))	77 (62.6)	102 (77.9)		179 (70.5)
**BMI z-score > 2 or 3 SD**				
Overweight (n (%))	21 (15.8)	40 (29)	0.009	61 (22.5)
Obese (n (%))	112 (84.2)	98 (71)		210 (77.5)
**Blood pressure** (**mmHg**)				
Within Normal range	121 (91)	132 (100)	0.04	253 (98.4)
With Hypertension	4 (9)	0 (0)		4 (1.6)
Median Systolic Blood Pressure (mmHg) (p25, p75)	110 (100, 120)	110 (110, 116)	NS	110 (100, 120)
Median Diastolic Blood Pressure (mmHg) (p25, p75)	70 (68, 80)	70 (60, 76)	0.002	70 (62, 80)
**Acanthosis Nigricans**				
Present	52 (41.9)	83 (63.8)	<0.001	135 (53.1)
Absent	72 (58.1)	47 (36.2)		119 (46.9)
**Metabolic Syndrome**				
No Metabolic Syndrome	116 (87.2)	127 (92)	NS	243 (89.7)
With Metabolic Syndrome	17 (12.8)	11 (8)		28 (10.3)

Missing data: 17 in pubertal status, 17 in acanthosis nigricans, 17 in insulin resistance.14 in blood pressure status NS: Not Significant.

**Table 2:** Anthropometric and Biochemical Measurements of 271 children.

	Boys	Girls	P value	All
Median BMI z-score (p25, p75)	2.9 (2.4, 3.3)	2.4 (1.9, 2.8)	<0.001	2.6 (2.1, 3)
Median Waist Circumference (cm) (p25, p75)	89 (83.2, 99.9)	85 (79.5, 90)	0.011	87 (81, 94)
Mean Body Fat (%)	38.7 ± 6.7	42.5 ± 4.7	<0.001	40.7 ± 6.1
**Biochemical Measurements**				
Median Fasting Plasma Glucose (mmol/l) (p25, p75)	5.3 (5, 5.5)	5.2 (4.3, 8.5)	NS	5.3 (5, 5.6)
Median HbA1c (%) (p25, p75) (mmol/mol)	5.3 (5, 5.5) 34 (31, 37)	5.2 (5, 5.5) 33 (31, 37)	NS	5.2 (5, 5.4) 33 (31, 36)
Mean Total Cholesterol (mmol/l)	4.6 ± 0.8	4.6 ± 0.8	NS	4.6 ± 0.8
Median Triglycerides (mmol/l) (p25, p75)	1.1 (0.8, 1.5)	0.9 (0.8, 1.3)	NS	1.0 (0.8, 1.4)
Median HDL-C (mmol/l) (p25, p75)	1.1 (1, 1.2)	1.1 (1, 1.3)	NS	1.1 (1, 1.3)
Mean LDL-C (mmol/l)	3.3 ± 0.9	3.2 ± 0.9	NS	3.2 ± 0.8
Median TG:HDL-C ratio (p25, p75)	2.1 (1.6, 2.99)	1.9 (1.5, 2.7)	NS	2.0 (1.5, 2.78)

NS: Not significant.

**Table 3:** Clinical characteristics of children with insulin resistance (IR) and with no insulin resistance (non-IR).

Clinical parameters	IR (n = 126)	Non-IR (n = 131)	p-Value
Mean Total Cholesterol (mmol/l)	4.6 ± 0.8	4.5 ± 0.8	0.09^a^
Median TG (mmol/l) (p25, p75)	1.1 (0.9, 1.4)	0.9 (0.7, 1.2)	0.001^b^
Median HDL-C (mmol/l) (p25, p75)	1.1 (1.0, 1.2)	1.1 (1.0, 1.3)	NS
Median LDL-C (mmol/l) (p25, p75)	3.2 (2.7, 3.8)	3.1 (2.5, 3.6)	0.04^b^
Median TG:HDL-C (p25, p75)	2.48 (1.6, 3.1)	1.73 (1.4, 2.6)	0.01^b^
Mean Systolic Blood Pressure (mmHg)	109.6 ± 10	109.5 ± 10	NS
Mean Diastolic Blood Pressure (mmHg)	69.7 ± 10	70.9 ± 8	NS
Median BMI z-score (p25, p75)	2.7 (2.3, 3.2)	2.5 (1.8, 2.9)	<0.001^b^
Median Waist Circumference (cm) (p25, p75)	89 (83.3, 96.5)	85 (79, 90)	<0.001^b^
Median Body Fat (%) (p25, p75)	42 (38.9, 46.3)	39.9 (35.2, 43.3)	<0.001^b^

NS: not significant.

^a^Independent sample t-test.

^b^Mann-Whitney U test.

**Table 4:** Correlation between TG:HDL ratio and other parameters of insulin resistance (IR).

	LDL	HDL	HOMA	BMI z-score	WC	TG:HDL-C
TC	0.931**	0.523**	0.091	0.014	0.000	−0.01
LDL		0.238**	0.081	0.081	0.039	0.05
HDL			0.039	−0.12*	−0.161*	−0.468**
HOMA				0.202**	0.258**	0.134*
BMI z-score					0.547**	0.104
WC						0.173**

TG: triglyceride, TC: total cholesterol, LDL: low-density lipoprotein, HDL: high-density lipoprotein, HOMA-IR: homeostasis model assessment of insulin resistance, BMI z-score: age and sex- corrected body mass index, TG:HDL-C ratio of TG to HDL. Correlation was estimated using Pearson correlation coefficient. ^*^P < 0.05, ^**^P < 0.000.

**Table 5:** Anthropometric, clinical and metabolic parameters across TG:HDL-C ratio tertiles.

	First tertile (25^th^ centile)	Second tertile (50^th^ centile)	Third tertile (75^th^ centile)	P value
n	90	91	90	
Age (y), mean (sd)	13.1 ± 2.3	13.1 ± 2.0	12.9 ± 1.9	NS
Male, n (%)	41	46	46	NS
Female, n (%)	49	45	45	NS
Pre-Pubertal, n (%)	21	28	26	NS
Pubertal, n (%)	65	54	60	NS
**Acanthosis Nigricans** (**n** (**%**))				
Present	39 (28.9)	38 (28.1)	58 (43)	0.005
Absent	46 (38.7)	45 (37.8)	28 (23.5)	
**Metabolic Syndrome** (**n** (**%**))				
No Metabolic Syndrome	85 (34.9)	86 (35.4)	72 (29.7)	0.001
With Metabolic Syndrome	5 (17.9)	5 (17.9)	18 (64.2)	
**Blood pressure** (**mmHg**) **n** (**%**)				
Within Normal range systolic and/or diastolic blood pressure	87 (34.4)	82 (32.8)	84 (32.8)	NS
With Hypertension	1 (16.7)	1 (44.4)	2 (38.9)	
BMI z-score, mean (sd)	2.4 ± 0.8	2.6 ± 0.8	2.8 ± 0.8	0.03
Waist Circumference (cm), mean (sd)	85.7 ± 10.3	88.4 ± 9.4	90.5 ± 9.7	<0.01
Body Fat Mass (kg), mean (sd)	25.5 ± 8.2	27.4 ± 8.4	28.7 ± 8.6	NS
Body Fat (%), mean (sd)	39.9 ± 5.8	40.9 ± 6.1	41.2 ± 6.3	NS
Systolic Blood Pressure (mmHg), mean (sd)	108 ± 10.4	110.3 ± 10.2	110.7 ± 10.9	NS
Diastolic Blood Pressure (mmHg), mean (sd)	69.8 ± 9.2	69.8 ± 10.8	71.7 ± 9.4	NS
Fasting Plasma Glucose (mmol/l), mean (sd)	5.3 ± 0.5	5.4 ± 0.5	5.7 ± 2.8	NS
Total Cholesterol (mmol/l), mean (sd)	4.5 ± 0.8	4.6 ± 0.7	4.6 ± 0.9	NS
TG (mmol/l), mean (sd)	0.7 ± 0.1	1.0 ± 0.2	1.6 ± 0.4	<0.001
HDL-C (mmol/l), mean (sd)	1.3 ± 0.2	1.1 ± 0.2	1.1 ± 0.2	<0.001
Mean LDL-C (mmol/l), mean (sd)	3.1 ± 0.9	3.3 ± 0.7	3.3 ± 1.0	NS
Mean TG:HDL-C ratio, mean (sd)	1.28 ± 0.27	2.1 ± 0.27	3.5 ± 1.0	<0.001
Mean Insulin (mU/mL), mean (sd)	15.1 ± 8	21.4 ± 29	22.0 ± 14.5	0.03
HOMA-IR, mean (sd)	3.6 ± 2.3	5.2 ± 7.5	6.1 ± 8.4	0.04

NS: Not significant.

**Table 6:** Associated factors of IR by simple and multiple logistic regression among obese and overweight students.

Variables	Univariate analysis			Multiple variable analysis		
	b	Crude OR (95% CI)	p-value	b	Adjusted OR (95% CI)	p-value
**TG:HDL-C**						
Tertile 1 (25^th^ percentile)	—	—	0.02	—	—	
Tertile 2 (50^th^ percentile)	0.62	1.87 (1.00, 3.47)	0.048	0.53	1.69 (0.85, 3.37)	0.14
Tertile 3 (75^th^ percentile)	1.1	2.99 (1.6, 5.58)	0.01	0.90	2.47 (1.23, 4.95)	0.01
Age	−0.25	0.78 (0.45, 1.32)	0.35	−0. 70	0.51 (0.15, 1.7)	0.28
Gender			0.624	−0.44	1.7 (0.93, 3.1)	0.09
Male	0.123	1.13 (0.69, 1.8)	—	—		
Female	−0.123	0.884 (0.54, 1.45)	—	—		
Pubertal stage			0.228	−0.13	0.88 (0.31, 2.49)	0.51
Pre-pubertal	0.333	1.395 (0.81, 2.4)	—	—		
Pubertal	−0.333	0.7 (0.42, 1.23)	—	—		
Waist circumference	0.07	1.07 (1.04, 1.1)	<0.001	0.081	1.08 (1.03, 1.13)	0.001
BMI-z score	0.66	1.93 (1.36, 2.75)	<0.001	0.007	1.00 (0.56, 1.8)	0.983

b = regression coefficients, OR = odds ratio, CI = confidence interval. Multivariate regression analysis was applied. Hosmer Lemeshow Test (chi-square = 5.56, p-value = 0.696) and classification table (overall correctly classified percentage = 67.5%).

## References

[b1] Kahn B. & Flier J. Obesity and insulin resistance. J Clin Invest. 106, 473–481 (2000). 10953022 10.1172/JCI10842 PMC380258

[b2] De Fronzo R. A., Tobin J. D. & Andres R. Glucose clamp technique: a method for quantifying insulin secretion and resistance. Am. J. Physiol. 237, E214–E223 (1979). 382871 10.1152/ajpendo.1979.237.3.E214

[b3] American College of Endocrinology: Insulin resistance syndrome (position, statement) *Endocr Pract*: **9**, 9–21 (2003). 12924350

[b4] Burke J. P., Hale D. E., Hazuda H. P. & Stern M. P. A quantitative scale of acanthosis nigricans. Diabetes Care. 22, 1655–1699 (1999). 10526730 10.2337/diacare.22.10.1655

[b5] Kim-Dorner S. J., Deuster P. A., Zeno S. A., Remaley A. T. & Poth M. Should triglycerides and the triglycerides to high-density lipoprotein cholesterol ratio be used as surrogates for insulin resistance? Metabolism 59, 299–304 (2010). 19796777 10.1016/j.metabol.2009.07.027

[b6] Hirschler V., Maccallini G., Sanchez M., Gonzalez Z. & Molinari C. Association between triglyceride to HDL-C ratio and insulin resistance in indigenous Argentinean children. Pediatric Diabetes. 16, 606–612 (2015). 25303644 10.1111/pedi.12228

[b7] Giannini C. . The triglyceride-to-hdl cholesterol ratio: association with insulin resistance in obese youths of different ethnic backgrounds. Diabetes Care. 34, 1869–1874 (2011). 21730284 10.2337/dc10-2234 PMC3142016

[b8] Sumner A. E., Finley K. B., Genovese D. J., Criqui M. H. & Boston R. C. Fasting triglyceride and the triglyceride-HDL cholesterol ratio are not markers of insulin resistance in African Americans. Arch Intern Med. 165, 1395–1400 (2005). 15983289 10.1001/archinte.165.12.1395

[b9] Kang H. T. . The association between the ratio of triglyceride to HDL-C and insulin resistance according to waist circumference in a rural Korean population. Nutr Metab Cardiovasc Dis. 12, 1054–1060 (2012). 10.1016/j.numecd.2011.01.013 21764572

[b10] Olson K., Hendricks B. & Murdock D. The triglyceride to HDL ratio and its relationship to insulin resistance in pre- and post-pubertal children: observation from the Wausau SCHOOL project. Cholesterol 2012, doi: 10.1155/2012/794252 (2012). PMC3395199 22811895

[b11] McLaughlin T. . Use of metabolic markers to identify overweight individuals who are insulin resistant. Ann Intern Med. 139, 802–809 (2003). 14623617 10.7326/0003-4819-139-10-200311180-00007

[b12] Hadaegh F. . Triglyceride/HDL-cholesterol ratio is an independent predictor for coronary heart disease in a population of Iranian men. Nutr Metab Cardiovasc. 19, 401–408 (2009). 10.1016/j.numecd.2008.09.003 19091534

[b13] National Health and Morbidity Survey 2011, Fact Sheet. Institute For Public Health, Ministry Of Health Malaysia.

[b14] Ferreira A. P., Oliveira C. E. & Franca N. M. Metabolic syndrome and risk factors for cardiovascular disease in obese children: the relationship with insulin resistance (HOMA-IR). J Pediatr (Rio J). 83, 21–6 (2007). 17183416 10.2223/JPED.1562

[b15] Lee S., Bacha F., Gungor N. & Arslanian S. A. Waist circumference is an independent predictor of insulin resistance in black and white youths. J Pediatr. 148, 188–94 (2006). 16492427 10.1016/j.jpeds.2005.10.001

[b16] Zimmet P., Alberti G., Kaufman F., Tajima N., Silink M., Arslanian S. . The metabolic syndrome in children and adolescents. Lancet. 369, 2059–61 (2007). 17586288 10.1016/S0140-6736(07)60958-1

[b17] Moreira Sérgio R. . Predicting insulin resistance in children: anthropometric and metabolic indicators. Jornal de pediatria. 84, 47–52 (2008). 18200334 10.2223/JPED.1740

[b18] Li C., Ford E. S., Meng Y. X., Mokdad A. H. & Reaven G. M. Does the association of the triglyceride to high-density lipoprotein cholesterol ratio with fasting serum insulin differ by race/ethnicity? Cardiovasc. Diabetol. 7 (2008). 10.1186/1475-2840-7-4 PMC2292689 18307789

[b19] Millán Jesús . Lipoprotein ratios: physiological significance and clinical usefulness in cardiovascular prevention. Vasc Health and Risk Manag. 5, 757–65 (2009). PMC2747394 19774217

[b20] Pacifico L. . Association of serum triglyceride-to-HDL cholesterol ratio with carotid artery intima-media thickness, insulin resistance and nonalcoholic fatty liver disease in children and adolescents. Nutr Metab Cardiovasc Dis. 24, 737–743 (2014). 24656140 10.1016/j.numecd.2014.01.010

[b21] Copeland K. C. . Characteristics of adolescents and youth with recent onset type 2 diabetes: the TODAY cohort at baseline. J Clin Endocrinol Metab. 96, 159–167 (2011). 20962021 10.1210/jc.2010-1642 PMC3038479

[b22] Rodriguez B. L. . Prevalence of cardiovascular disease risk factors in US children and adolescents with diabetes: the SEARCH for diabetes in youth study. Diabetes Care. 29, 1891–1896 (2006). 16873798 10.2337/dc06-0310

[b23] Goran M. I. . Impaired glucose tolerance and reduced β-cell function in overweight Latino children with a positive family history for type 2 diabetes. J Clin Endocrinol Metab. 89, 207–212 (2004). 14715851 10.1210/jc.2003-031402

[b24] Lee S., Bacha F., Gungor N. & Arslanian S. A. Waist circumference is an independent predictor of insulin resistance in black and white youths. J Pediatr. 148, 188–194 (2006). 16492427 10.1016/j.jpeds.2005.10.001

[b25] Freedman D. S., Khan L. K., Dietz W. H. & Berenson G. S. Relationship of childhood obesity to coronary heart disease risk factors in adult-hood: the Bogalusa Heart Study. J Pediatr 108, 712–718 (2001). 10.1542/peds.108.3.712 11533341

[b26] Juonala M. . Risk factors identified in childhood and decreased carotid artery elasticity in adulthood: the cardiovascular risk in youth Finns study. Circulation. 112, 1486–1493 (2005). 16129802 10.1161/CIRCULATIONAHA.104.502161

[b27] Visser M., Bouter L. M., McQuillan G. M., Wener M. H. & Harris T. B. Low-grade systemic inflammation in overweight children. J Pediatr. 107, E13–E13 (2001). 10.1542/peds.107.1.e13 11134477

[b28] He S. . Higher ratio of triglyceride to high-density lipoprotein cholesterol may predispose to diabetes mellitus: 15-year prospective study in a general population. Metab. Clin. Exp. 61, 30–36 (2012). 21664631 10.1016/j.metabol.2011.05.007

[b29] Marshall W. A. & Tanner J. M. Variations in pattern of pubertal changes in girls. Arch Dis Child. 44, 291–303 (1969). 5785179 10.1136/adc.44.235.291 PMC2020314

[b30] Marshall W. A. & Tanner M. Variations in pattern of pubertal changes in boys. Arch Dis Child. 45, 13–23 (1970). 5440182 10.1136/adc.45.239.13 PMC2020414

[b31] Organization WH. Training Course on Child Growth Assessment- WHO Child Growth Standards (2008).

[b32] Reinehr T. & Andler W. Changes in the atherogenic risk factor profile according to degree of weight loss. Arch. Dis. Child 89, 419–422 (2004). 15102630 10.1136/adc.2003.028803 PMC1719907

[b33] Alberti K. G. M. M., Zimmet P. Z. & JE S. The metabolic syndrome in children and adolescents. Lancet. 369, 2059–2061 (2007). 17586288 10.1016/S0140-6736(07)60958-1

[b34] Poh B. K. . Waist circumference percentile curves for Malaysian children and adolescents aged 6.0–16.9 years. Pediatr Obes. 6, 229–235 (2011). 10.3109/17477166.2011.583658 21668385

[b35] Rosner B., Prineas R. J., Loggie J. M. & SR D. Blood pressure nomograms for children and adolescents, by height, sex and age in the United States. J Pediatr. 123, 871–86 (1993). 8229519 10.1016/s0022-3476(05)80382-8

